# Cerclage outcome by the type of suture material (COTS): study protocol for a pilot and feasibility randomised controlled trial

**DOI:** 10.1186/1745-6215-15-415

**Published:** 2014-10-27

**Authors:** Fidan Israfil-Bayli, Philip Toozs-Hobson, Christoph Lees, Mark Slack, Khaled Ismail

**Affiliations:** Birmingham Women’s Hospital NHS Foundation Trust, Edgbaston, Birmingham, B15 2TG UK; Urogynaecology Department, Birmingham Women’s Hospital NHS Foundation Trust, Edgbaston, Birmingham, B15 2TG UK; Imperial Centre for Fetal Care, Queen Charlotte’s and Chelsea Hospital, Imperial College Healthcare NHS Trust, London, W12 0HS UK; Urogynaecology Department, Addenbrooke’s Hospital, Cambridge University Hospitals NHS Foundation Trust, Cambridge, CB2 2QQ UK; School of Clinical & Experimental Medicine, College of Medical & Dental Sciences, University of Birmingham, Edgbaston, Birmingham, B15 2TT UK

**Keywords:** Cervical cerclage, Live birth, Preterm birth, Multifilament sutures, Monofilament sutures

## Abstract

**Background:**

Cervical incompetence is one of the causes of preterm birth and mid-trimester pregnancy loss. Cervical cerclage is a surgical procedure to treat cervical incompetence. Cervical cerclage reduces the incidence of preterm birth in women at risk of recurrent preterm birth, without a statistically significant reduction in perinatal mortality or neonatal morbidity. Multifilament/braided sutures such as Mersilene tape have been traditionally used for cervical cerclage. Braided sutures, particularly mesh-like non-absorbable sutures, have been associated with an increased risk of infection and, hence, some obstetricians prefer to use monofilament/non-braided sutures. However, these claims are not substantiated by any scientific or clinical evidence.

We propose a pilot/feasibility study which will provide the necessary information for planning a definitive trial investigating the clinical effectiveness of monofilament non-braided suture materials in reducing pregnancy loss rate following cervical cerclage compared to the traditional multifilament braided sutures.

**Methods/Design:**

Women eligible for elective or ultrasound-indicated cerclage at 12 to 21 + 6 weeks of gestation will be randomised to having the procedure using either a monofilament non-braided suture (Ethilon) or a Multifilament braided suture (Mersilene tape) inserted using a McDonald technique. Consent for participation in the Cerclage outcome by the type of suture (COTS) study will be obtained from each eligible participant.

**Clinical trials registration:**

COTS is registered with the International Standard Research for Clinical Trials (ISRCTN17866773). Registered on 27 March 2013.

## Background

Cervical incompetence is one of the important causes of prematurity for which cerclage has been used for many years. A recent Cochrane review showed a trend to a reduction in neonatal death and neonatal morbidity; neither alone was significant [1]. None of the studies included in the Cochrane review addressed the question of the type of suture material, an important determinant of outcomes of surgical procedures in general. This issue is of particular relevance because Mersilene tape, the traditionally used surgical material for cerclage, has been associated with an increased risk of infection in other surgical disciplines [2, 3]. Indeed, infection is an important underlying cause for failed cerclage and preterm labour [4]. It is for this reason that some surgeons use monofilament suture material, although this is not evidence-based. However, the strength of such suture material and the risk of it traumatising cervical tissue is a concern for some, albeit an unsubstantiated one. We conducted a national survey of Obstetrics and Gynaecology consultants in the UK, which confirmed variability in practice - the majority of respondents were using multifilament/braided sutures, whereas only 16.6% used monofilament non-braided sutures. Significantly, 75% of respondents stated that there was no guidance for which suture material to use within their unit [5]. We subsequently conducted a systematic review, which identified only two non-randomised studies (NRS). The NRS meta-analysis demonstrated that non-braided cervical suture is associated with a significant risk reduction in pregnancy loss compared to multifilament/braided sutures (odds ratio =0.24; 95% confidence interval 0.06 to 0.96) [6].

We hypothesised that the use of monofilament sutures is associated with reduced risk of foetal loss after cerclage. The aim of this publication is to present the research protocol of a pilot/feasibility study which will provide the necessary information for planning a definitive trial investigating the clinical effectiveness of monofilament non-braided suture materials in reducing pregnancy loss rate following cervical cerclage compared to the traditional multifilament braided sutures. Thus Cerclage outcome by the type of suture (COTS) is a feasibility/pilot randomised controlled trial (RCT) to inform a number of aspects of how the definitive trial may be optimally delivered.

### Strengths and weakness of this study

There are several limitations to the proposed study: the lack of robust prospective data to undertake a formal sample size calculation; unavailability of information about the level of clinician’s engagement; or information about expected recruitment or attrition rates. Therefore, we designed this as a pilot feasibility study to provide the information that will be essential for a future full-scale trial.

In contrast, there are several strengths to this study including the novelty of the research question, the availability of plausible mechanistic hypotheses and the prioritisation of the research question by the majority of clinicians responding to the national review of practice in relation to cerclage procedures. Moreover, members of the Royal College of Obstetricians and Gynaecologists Preterm birth clinical study group and the maternity-specific patient and public involvement group were involved in the study design and determining outcomes of relevance.

## Methods/design

**Figure 1:**
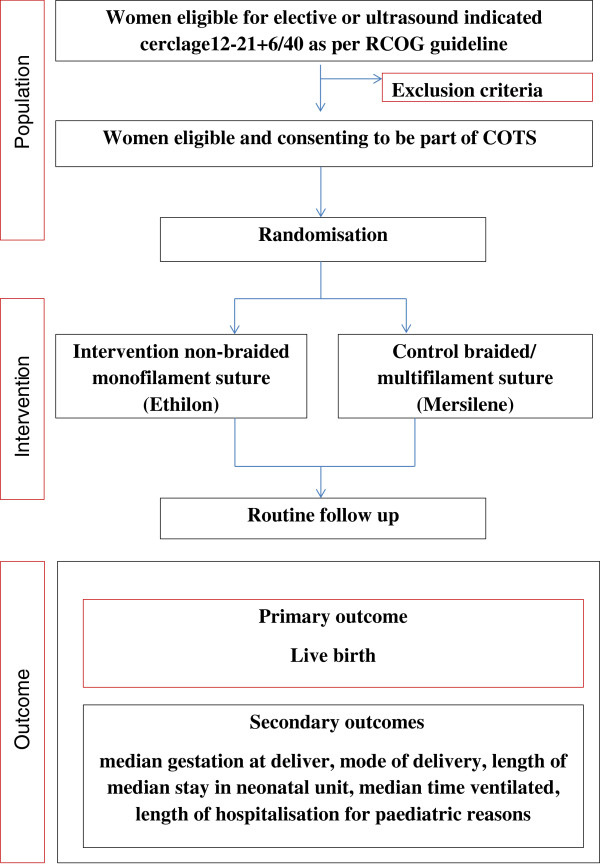
**Study flow chart.** COTS, Cerclage outcome by the type of suture; RCOG, Royal College of Obstetricians and Gynaecologists.

COTS is a pilot and feasibility RCT comparing monofilament (intervention) sutures versus multifilament (comparison) for cervical cerclage (Figure 1).

The study will provide an opportunity to prepare for the challenges and difficulties within a definitive study. We are testing the study protocol and planning to facilitate a formal sample size calculation for the larger RCT.

The consent for participation in the trial will be obtained from each participant.

### Ethical approval

The study has been approved by the Ethics Committee (Edgbaston Research Ethics Committee). The study has been approved by Research and Development Departments in each of the three Units (Birmingham Women’s Hospital NHS Foundation Trust, Addenbrooke's Hospital, Sandwell and West Birmingham NHS Trust).

### Setting

The pilot and feasibility RCT is conducted in three maternity centres in the UK in order to assess likely recruitment rates and acceptability across different sites.

### Study population and eligibility criteria

Participants consist of women eligible for elective or ultrasound-indicated cerclage 12 to 21 + 6 weeks of gestation as per Royal College of Obstetricians and Gynaecologists guideline [4].

### Inclusion criteria

Singleton pregnancies in women:

- With a history of three or more previous mid-trimester losses or premature (<28 week) births
- Who have had cervical sutures in previous pregnancies
- With history of mid-trimester loss or premature birth with a shortened cervix (<25 mm)
- Who are deemed at risk of preterm birth at the clinician's discretion

### Exclusion criteria

- <18 years old
- Multiple pregnancy
- Women who are unable to give informed consent
- Women unwilling to consent to the study

### Consent and randomisation

The study will be discussed with the women and they will be provided with an information leaflet ensuring they have enough time to read it and discuss it, as cerclage is usually booked as a scheduled procedure on a separate day.

Patients are reassured about confidentiality, are advised that declining participation will not affect their clinical care, and told that they can withdraw the consent at any point in the pathway. To reduce the potential for bias, computer or telephone randomisation occurs immediately before surgery. The allocation ratio will be 1:1; randomisation is in blocks.

### Intervention

The monofilament non-braided suture used is Ethilon®, (Ethicon, UK) and the multifilament braided suture is Mersilene® tape (Ethicon, UK) for the intervention and control groups, respectively. The stitch is inserted using a McDonald technique. Planned removal of the suture would occur at 37 (±1) weeks of gestation. When the stitch is removed it is retained for microbiological investigations. A vaginal swab is taken before the cerclage procedure to ensure that any infection is treated before inserting the suture. A vaginal swab will also be taken at the time of suture removal. The follow-up should not involve additional contact with the patient beyond routine local clinical management protocols. The study will be deemed complete when the last recruited woman has delivered and, if applicable, her baby is discharged from the neonatal unit.

In view of logistic difficulties in ensuring blinding of participants, and the fact that all the COTS outcomes are objective measures that would be easily and independently retrieved from hospital records (and hence it is unlikely that the lack of blinding will be a cause of serious bias), we do not intend to attempt blinding participants or assessors.

### Study outcome measures

#### Primary outcome

Live birth rate

#### Secondary outcomes

- Median gestation at delivery
- Mode of delivery
- Length of median stay in neonatal unit
- Median time ventilated
- Length of hospitalisation for paediatric reasons

### Withdrawal from COTS study

Participants may withdraw from the study at any time. Should they choose to withdraw, they will continue to be followed up, in line with current practice within the participating unit.

### Safety monitoring procedures

Within the COTS trial, a serious adverse event (SAE) is defined as an untoward occurrence:

- Miscarriage/preterm labour within 24 hours of cerclage
- Premature rupture of membranes within 48 hours
- Prolongation of existing hospitalisation
- Significant haemorrhage, requiring blood transfusion
- Infection requiring intravenous antibiotics
- Life-threatening conditions
- Is otherwise considered medically significant by the investigator

### Reporting serious adverse events

SAEs believed to be due to surgery (cervical cerclage) should be reported on a Serious Adverse Event form and faxed to the COTS study office (this should be the sponsor or Chief Investigator (CI)). SAEs still present at the end of the study must be followed up at least until the final outcome is determined, even if it implies that the follow-up continues after the patient finishes the study treatment and, when appropriate, until the end of the planned period of follow-up. The CI will report all SAEs to the Data Monitoring Committee approximately 3-monthly, to the main Research Ethics Committee annually, and to the Trial Steering Committee 6-monthly. Local Investigators are responsible for reporting SAEs to their host institution, according to local regulations, but they do not need to inform the main Research Ethics Committee as this will be done by the CI.

### Sample size

The size of the pilot study will not allow reliable assessment of the effect of the intervention on clinical outcomes and so hypothesis testing is not proposed.

In the pilot study, analyses will principally take the form of simple descriptive statistics of process outcomes, including eligibility and recruitment rates, and of live birth rate and secondary clinical outcomes, to aid designing of the main trial. Regression models, appropriate to the forms of data, will also be fitted, to allow adjustment for covariates.

During the pilot study, accurate assessment of the numbers of women eligible, approached and recruited will be made. The systems and data collection tools will be developed and piloted. These include the telephone randomisation system and the collection of the clinical data from both woman and baby prior to discharge from hospital.

## Discussion

We have initiated a pilot/feasibility trial to provide important information to plan and confidently run a definitive trial that will be able to determine the clinical and cost effectiveness of the different types of suture material. We are aiming to achieve several objectives:

- To test the study protocol that has been designed with the full scale trial in mind
- Test recruitment and randomization procedures
- Estimate rate of recruitment and attrition in order to plan the scale and duration of the definitive study
- Examine general data collection, cleaning, input and analysis procedures
- Establish the benefits of the one type of suture over the other in cases of elective cervical cerclage.

We will aim to recruit eligible women who agree to participate in recruiting centres over a 12-month period. Undoubtedly, the size of the pilot study will not allow reliable assessment of the effect of the different suture materials on clinical outcomes. However, this is not one of the aims of this study. Nevertheless, we will collect information from collaborating units on the numbers of women requiring cervical cerclage and who are eligible for the study and the birth outcomes of those women. This will give a larger prospective sample on which to base the power calculation.

Based on our NRS meta-analysis, the pregnancy loss rate was 4% and 16% in the multifilament/braided versus monofilament/non-braided groups, respectively. In view of the small sample size and lack of randomisation it is highly likely that this effect size is exaggerated. Based on 49 cases of cerclage using non-braided sutures undertaken in two large maternity units, the pregnancy loss rate in this cohort was 5%. In contrast, in the cerclage group of RCTs included in the Cochrane review where braided sutures were used as standard, the cumulative pregnancy loss rate was 13%. Therefore, to demonstrate a more reserved reduction in this risk from 13 to 5% with the use of monofilament sutures we will require 267 women in each group (a total of 534 women) with 90% power (*P* =0.05). To allow for a 10% loss to follow-up we have increased the total sample size to 600. Assuming a decline rate of 25%, we will need to approach 800 eligible women to achieve the required sample size for a full-scale trial to show similar levels of pregnancy. We are fully aware that the proposed sample size will be reviewed in light of the results of the pilot study.

If the hypothesised benefit of monofilament sutures is confirmed, this policy will be rapidly adopted nationally. In the UK alone this could potentially prevent more than 350 babies per annum dying as a result of mid-trimester loss, intrauterine infection or complications of prematurity. Moreover, reducing the risk of prematurity will reduce neonatal unit and hospital stay, the significant morbidity associated with early gestation, and the associated long-term morbidity.

## Trial status

Recruitment is ongoing at the time of submission.

## Abbreviations

CI: Chief Investigator
COTS: Cerclage outcome by the type of suture
NRS: non-randomised studies
RCT: randomised controlled trial
SAE: serious adverse event.
